# Application of Adaptive and Switching Control for Contact Maintenance of a Robotic Vehicle-Manipulator System for Underwater Asset Inspection

**DOI:** 10.3389/frobt.2021.706558

**Published:** 2021-07-28

**Authors:** Kamil Cetin, Carlos Suarez Zapico, Harun Tugal, Yvan Petillot, Matthew Dunnigan, Mustafa Suphi Erden

**Affiliations:** ^1^ Heriot-Watt University, Institute of Sensors, Signals, and Systems, Edinburgh, United Kingdom; ^2^ Edinburgh Centre for Robotics, Edinburgh, United Kingdom

**Keywords:** surface inspection, force/position control, admittance control, contact interaction, underwater vehicle and manipulator system

## Abstract

The aim of this study is to design an adaptive controller for the hard contact interaction problem of underwater vehicle-manipulator systems (UVMS) to realize asset inspection through physical interaction. The proposed approach consists of a force and position controller in the operational space of the end effector of the robot manipulator mounted on an underwater vehicle. The force tracking algorithm keeps the end effector perpendicular to the unknown surface of the asset and the position tracking algorithm makes it follow a desired trajectory on the surface. The challenging problem in such a system is to maintain the end effector of the manipulator in continuous and stable contact with the unknown surface in the presence of disturbances and reaction forces that constantly move the floating robot base in an unexpected manner. The main contribution of the proposed controller is the development of the adaptive force tracking control algorithm based on switching actions between contact and noncontact states. When the end effector loses contact with the surface, a velocity feed-forward augmented impedance controller is activated to rapidly regain contact interaction by generating a desired position profile whose speed is adjusted depending on the time and the point where the contact was lost. Once the contact interaction is reestablished, a dynamic adaptive damping-based admittance controller is operated for fast adaptation and continuous stable force tracking. To validate the proposed controller, we conducted experiments with a land robotic setup composed of a 6 degrees of freedom (DOF) Stewart Platform imitating an underwater vehicle and a 7 DOF KUKA IIWA robotic arm imitating the underwater robot manipulator attached to the vehicle. The proposed scheme significantly increases the contact time under realistic disturbances, in comparison to our former controllers without an adaptive control scheme. We have demonstrated the superior performance of the current controller with experiments and quantified measures.

## 1 Introduction

The demand for underwater vehicle-manipulator systems (UVMS) has been increasing rapidly in the offshore industry in recent years. One of the most important developments in this area is the emergence of autonomous UVMS for underwater interaction (Cataldi and Antonelli, 2015; Dhanak and Xiros, 2016; Kim et al., 2016; Sivčev et al., 2018). Subsea tasks such as underwater welding, surface scanning, corrosion detection, and valve turning, which are dangerous, expensive, and made by humans under difficult conditions, can be done more easily, cheaply, and safely with UVMS. A major challenge in such applications is to keep the end effector of UVMS in contact with the underwater asset at all times despite the disturbing effects in the water. This problem can actually be considered as a common problem to all kinds of mobile-based robot manipulators that move on land, aerially, or under water.

In these applications, the surface structure and its 3D model are usually unknown to the robotic system. The end effector of the underwater manipulator needs to be held perpendicular to the surface in as much continuous contact as possible, despite the disturbing effects. In this study, we specifically focus on the problem of keeping the end effector in contact on the surface as much as possible. There are two main causes for the end effector of the system to lose contact with the surface. The first cause is the water currents and waves that disturb and move the base vehicle resulting in the manipulator and its end effector detachment from the surface. The second cause is the reaction forces from the surface structure to the end effector during the contact and predominantly at the moment that contact is achieved. This happens as a result of the base vehicle initially pushing the manipulator and its end effector towards the surface to achieve contact and to maintain a constant force level and then the reaction force pushing the overall system back as well as the resulting large interaction forces causing the controller to make a sudden backward movement that overall results in loss of contact. When the end effector loses contact with the surface, the controller needs to react very quickly to reestablish contact with the surface, and if the end effector is already in contact with the surface, the controller should maintain the contact force continuously at the desired force level; in other words, the controller needs to handle two different situations. To address this challenge, in this study, we explore the adaptive and switching control techniques based on an admittance controller and develop an adaptive-switching-admittance controller that provides promising performance to maintain continuous contact under the disturbance conditions recorded from a realistic vehicle movement under water.

An overview of force contact interaction methods can be found in (Boissonnat et al., 2004), (Antonelli, 2014). In the early studies on the force contact interaction of UVMS (Antonelli et al., 1999; Antonelli et al., 2001), the authors presented a task-priority inverse kinematic redundancy resolution-based force control method to reduce possible contact loss of UVMS. In the study (Cui and Sarkar, 2000), a fuzzy switching-based hybrid force/position controller was designed for UVMS. However, a simple PID controller is used for force control in the aforementioned studies. In (Olguín-Díaz et al., 2013) and (Heshmati-Alamdari et al., 2018), the authors proposed a force/position control approach with a task-priority-based redundancy method where contact force trajectories for the end effector were defined as the primary task and a posture of the UVMS as the secondary tasks. In (Barbalata et al., 2018) and (Razzanelli et al., 2019), the authors designed the force/position controllers for the contact interaction of the end effector of UVMS with environment. In (Cieslak and Ridao, 2018), an admittance control (position-based impedance control) was developed for contact force control of UVMS. However, all aforementioned studies focused on the task-priority redundancy resolution of UVMS and did not consider the disturbance effects of the base vehicle movement on the force contact interaction as they all aimed directly at controlling the position of the end effector.

In the literature, the hard contact interaction is a fundamental problem for all types of mobile-based robot manipulators. The authors in (Mersha et al., 2014) presented a variable impedance controller for the aerial force interaction. In the hybrid model predictive control method for physical interaction of aerial robots (Alexis et al., 2016), the force trajectory controller depended on the varying force reference. In (Ryll et al., 2019), they proposed an admittance controller for the physical interaction of aerial robots by estimating the interaction forces. However, all these controllers require the position data of the interacted environment and use a force estimation rather than direct measurements. In our study, we develop a controller to adapt to unknown (no position data) surfaces through exploiting the force/torque interaction and use direct force/torque measurements by a sensor.

In Section 2, we present the background and problem definition of the force interaction problem for mobile manipulators with the related force control techniques that we adopt such as the mas-damping admittance controllers. In Section 3, we describe the properties of the admittance controllers and how the parameter tuning can affect the system response and then analyze how the system evolves in the case of a sudden contact loss. In subsections, we propose the switching algorithm for the position control to handle the contact loss. In Section 4, we present the experimental setup for the simulation and the real robot system, and in Section 5, we give the results obtained from our experiments. Section 6 concludes the paper.

## 2 Background and Problem Definition

Force tracking with mobile manipulators in underwater vehicles is an application subject to a lot of uncertainties that need to be adapted on the go. There are not only structured uncertainties like the contact dynamics when interacting with high-stiffness environments and how the mass distribution affects the motion dynamics of the robotic system but also unstructured uncertainties such as hydrodynamic effects, disturbances, or complex and highly nonlinear dynamics. This means that high adaptability and robustness are required to deal with all these uncertainties and to extend the range of conditions that the controller can work properly.

In the underwater tasks that we target, there is limited access to observations and prior knowledge of the system dynamics. The only observations available are the force readings from the force/torque sensor attached at the manipulator end effector and the position data coming from the joints of the manipulator. Additionally, a motion model of the underwater vehicle is usually not available, and this information cannot be integrated into the controller to make predictions or identify the properties of the environment. The lack of these observations and prior knowledge of the motion dynamics narrows down the available techniques that can be used, forcing us to search for a robust model-free adaptive controller with a good and fast reaction control to compensate for unpredictable disturbances from the environment. Also, for this application, high precision force tracking is not crucial, but force/torque sensing is required to monitor the interaction with the surface to maintain continuous contact, reduce the contact losses, and guarantee the stability and the integrity of the robotic platform, which can be damaged due to high peak forces.

There are two major trends of interaction control that can be followed, direct and indirect interaction control (Villani and De Schutter, 2008). Using direct force control has the advantage of performing faster and more accurate force control optimized for the interaction conditions, but it is also much less robust against uncertainties. On the other hand, indirect interaction, which we adopt here, is less accurate to follow the desired force reference, but it is more robust when interacting in a wider set of conditions and less prone to running into instability when interacting with a hard contact.

Indirect interaction can be applied in a robot using an impedance or admittance causality with the environment. Admittance control tends to have a better performance in free motion or when interacting with very compliant or viscoelastic materials. In underwater manipulation, for example, the robot moves through the water, which is about 50 times more viscous and 1,000 times more dense compared to air, so we can consider the water to represent a soft environment in which admittance control would perform better than impedance. In (Ott et al., 2010), a hybrid admittance and impedance controller is implemented to benefit from the advantages of each controller, and the authors obtained a system that has a better behavior in a broader set of conditions imposed by the environment.

When there is interaction with unknown environments, adaptability in the controller allows tuning its parameters and gains in order to achieve certain goals, such as a transitory behavior, reduced contact loss time, ensuring the passivity of the system, or extending the range of conditions in which the system can operate. One common approach known as indirect adaptive control is to make an online estimation of the properties of the environment from the force-position data pairs during the interaction and update the control gains based on these estimations (Haddadi and Hashtrudi-Zaad, 2008). Another major branch in adaptive control is the direct adaptive control or Model Reference Adaptive Control (MRAC) (Coman and Boldisor, 2014), where a reference model that defines the desired transitory and steady-state behavior in the coupling dynamics is given. The MRAC controller then tries that the closed-loop response matches the reference model. Other approaches also include energy-aware controllers that compute the energy emitted and absorbed from the interaction port to maintain the passivity of the controller (Stramigioli et al., 2015), (Schindlbeck and Haddadin, 2015), and (Hamaza et al., 2019), the optimization of a predefined cost function (Matinfar and Hashtrudi-Zaad, 2004) and (DiMaio et al., 2004), or even model predictive control (de la Casa Cárdenas et al., 2015) and (Baptista et al., 2006). The fact that we do not have knowledge on how the robot is moving relative to the environment or vice versa jeopardizes fully monitoring the environment identification, monitoring the energy flow between the environment and robot, and applying any prediction for compensation and even poses problems for computing the cost of changing the controller gains in the optimization of a predefined cost function. This is why we are forced to search for a model-free adaptive control with a good disturbance rejection depending only on the force error feedback.

The stages of an interaction in such an application can be divided into the free motion phase, the impact phase designating the instant of getting into contact, and the constrained motion phase. Some approaches (Zotovic Stanisic and Valera Fernández, 2009) use different control structures or parameter switching for each phase, while others (Chiaverini and Sciavicco, 1993; Carloni et al., 2007) have only two, one for free and another for constrained motion. There is also the possibility of using one control structure for the three phases like in the case of the mass-spring-damper impedance control.

The impact is the most critical phase for the stability and integrity of the equipment. In some approaches (Stanisic and Fernández, 2012), a specific control structure is used separately for this phase. The technical challenge of the impact management comes from the fact that it is an extremely brief event, which may last for a few sampling periods and that, for an adaptive controller, it is too fast to identify the anomaly and react on time with optimized parameter gains. A specific impact controller would try to dissipate the kinetic energy and smoothen the impact rather than focusing on regulating the system to the force reference. As an example in (Stanisic and Fernández, 2012), the authors designed a new controller that applies a sliding mode controller during the impact phase in order to quickly dissipate the energy and reduce the risk of damaging effects from the high force peaks and then switching to a constrained motion control structure to track the signal reference. Other strategies or factors that can smoothen the impact are reducing the speed when the impact is expected as well as reducing the virtual inertia to limit the kinetic energy and augment the virtual damping to dissipate that energy once contact takes place.

One of the most extended model-free controllers for interaction is the mass-damping admittance control, and given the specifications in our application, it is also a good candidate because of its passive nature which provides robustness against a wide range of environments. A model-free adaptive admittance controller to improve the disturbance rejection was introduced in (Jung and Hsia, 1999), (Jung et al., 2004), and (Duan et al., 2018) and denominated as “adaptive hybrid impedance control,” which from now on we refer to as AAC. AAC shows a more accurate force tracking than a fixed-gain admittance control, but it also shows a worse impact response, generating higher overshoots which result in more oscillations and more risk of contact losses. A modification of AAC is presented in (Cao et al., 2019) and (Cao et al., 2020), named by the authors as “dynamic adaptive hybrid impedance” and denoted in our paper as DAAC. The overall idea of DAAC is to have a hybrid response of the admittance controller during the impact phase and transitions and preserve the AC response in the steady state. In this study, we adapt these DAAC and AAC controllers and compare them to the switching dynamic adaptive admittance controller that we develop and introduce in the subsequent sections.

**FIGURE 1:**
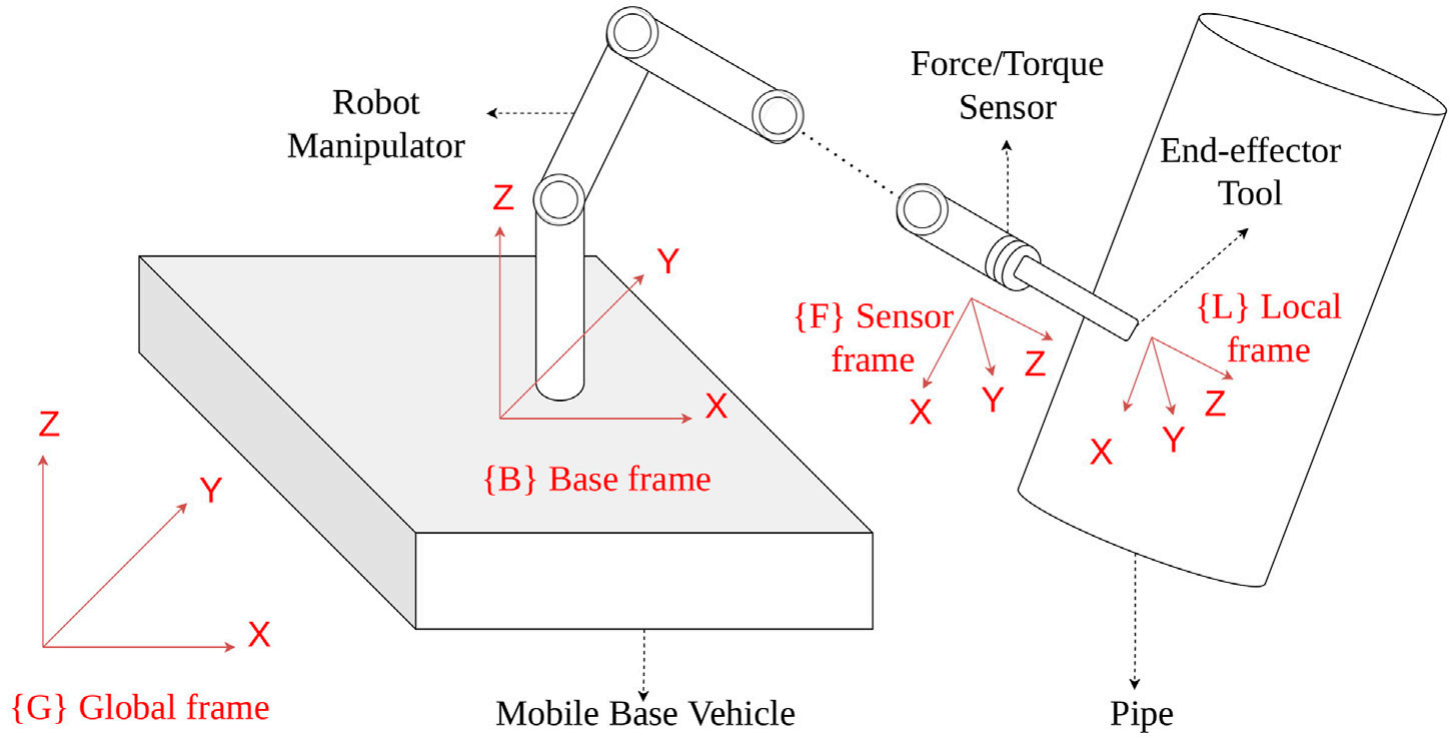
Illustration of the end effector attached with a force sensor of the mobile-based manipulator interacting with a pipe in the presence of disturbance effects on the base vehicle.

The problem can be defined as developing a controller for a system where the end effector of an *n* degrees of freedom (DOF) mobile-based manipulator remains in constant contact with a (pipe) surface and then a trajectory tracking motion is performed in the end-effector space to move the end effector on the pipe surface while an *m* DOF base vehicle motion is disturbed by hydrodynamic and reaction forces. Figure 1 illustrates a typical application scenario with the reference frames of a mobile-based manipulator with a force sensor at its end effector, attached to a moving base vehicle and interacting with a pipe. When contact between the end effector and the pipe surface is achieved, a certain trajectory tracking motion is to be performed tangentially on the pipe surface.

## 3 Hybrid Force/Position Control Architecture

**FIGURE 2:**
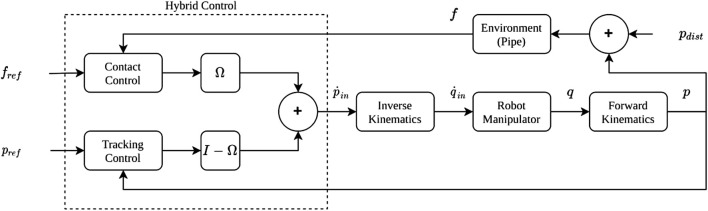
Block diagram of the hybrid control architecture.

The hybrid force/position control architecture is presented in this section (Figure 2). Taking into account the unknown disturbance effect of the base vehicle to the position of the robot manipulator, the proposed control architecture is enhanced *via* an admittance control approach in Section 3.1. For tracking of a trajectory on an unknown 3D surface, in Section 3.2, we have directly adapted our previous work (Moura et al., 2018), where an operational space control was performed with a PD controller to clean the surface of an unknown 3D surface with a fixed based robot manipulator. In the current paper, we will not explain this approach in detail as the reader can refer to (Moura et al., 2018) for the details, testing, and verification of the method. In the current paper, our focus will be maintaining contact with the structure, in order to allow the controller in (Moura et al., 2018) to operate properly with the floating robot base.

### 3.1 Control Design for Surface Contact

#### 3.1.1 Admittance Controller

The core structure of the constrained motion controller is a virtual mass-damping admittance control with no virtual stiffness component, as there is no knowledge of the surface structure and shape. In order to track a desired force reference $f_{ref}$ with zero errors in the steady state, a mass-damping admittance controller can be defined as

$$f_{ref}-f=M\ddot{p}+D\dot{p}$$ (1)

where the virtual mass is denoted by *M*, damping by *D*, the measured force by *f*, and the calculated end-effector position by *p* from the measured joint positions. Expecting interactions with high-stiffness environments, the controller should be tuned to have an overdamped response required to compensate for the underdamped interaction behavior in order to prevent high peak forces in the impact phase as well as contact losses due to bouncing off the surface induced from those force peaks. The manipulator should be controlled to respond as the dual of the environment. An overdamped response can be obtained by setting high virtual damping to dissipate the energy of the impact and a low virtual mass to reduce the inertia and kinetic energy. An overdamped controller is beneficial during the impact phase but it also makes the system have a slow reaction and perform worse at disturbance rejection, resulting in a less accurate force tracking controller. And admittance controller with fixed gains is inherently stable and a stable interaction can be assured as long as the environment remains passive.

#### 3.1.2 Dynamic Adaptive Admittance Controller

When an AC controller is set to behave as an overdamped system to counteract high-stiff contacts and prevent high impact forces, disturbance rejection and reference following performance are badly affected. By adding a variable damping component *ρ* (Eq. 2) which is updated with the adaptation law in expression (3), where the parameter *σ* is set to a fixed value, the system response to disturbances improves due to a faster reaction. However, this also comes with an overshoot response during the impact phase which can induce harmful peak forces. The DAAC implements a hybrid solution to combine the best of both controllers, the overdamped response in AC for the transitory phase, and the superior disturbance rejection during the steady state that the variable damping with fixed *σ* provides. DAAC is able to generate this hybrid behavior by using an additional adaptation law, defined in Eq. 4, which returns a variable *σ*. There are three parameters to tune, *α, β,* and $\sigma_{max}$. $\sigma_{max}$ is the maximum value that (4) returns when the system is in a perfect steady state (Δf=0 and $\dot{\text{Δ}f}=0$). In (Jung and Hsia, 1999) and (Jung et al., 2004), the authors mathematically demonstrated that, in order to guarantee the stability of the controller, *σ* should follow the constraint defined in (5) where *T* is the sampling time of the controller. However, the theoretical maximum value is not necessarily the optimum value for this parameter; therefore, an *ad hoc* selection of $\sigma_{max}$ is done experimentally with the real experimental setup to give the best performance possible in our trials.

$$f_{ref}-f=M\ddot{p}+D\left(\dot{p}+\rho \left(t\right)\right)$$ (2)

$$\rho \left(t\right)=\rho \left(t-T\right)+\sigma \frac{\left(f_{ref}\left(t-T\right)-f_{e}\left(t-T\right)\right)}{D}$$ (3)

$$\sigma =\frac{1}{\left(e^{\alpha |\text{Δ}f|}-1\right)+\left(e^{\beta |\dot{\text{Δ}f|}}-1\right)+\frac{1}{\sigma_{max}}}$$ (4)

$$0<\sigma <\frac{DT}{DT+M}$$ (5)

**FIGURE 3:**
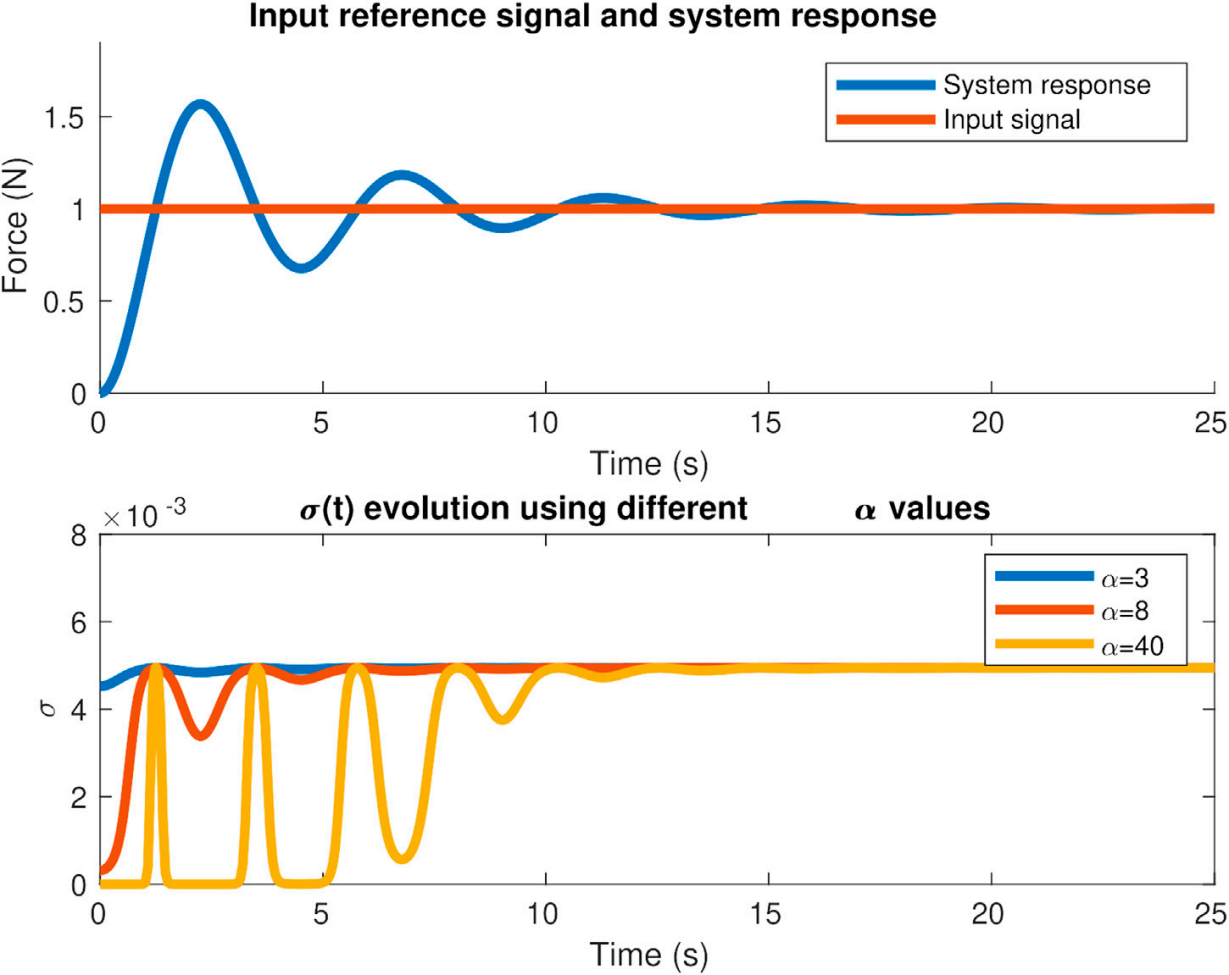
Effect of *α* on *σ* evolution for a second-order system response to a desired step force signal.

The *α* parameter works as a proportional gain to the force error and due to the nonlinearity of the adaptation law in (4), the optimal value is dependent not only on the properties of the environment but also on the desired force to apply. Figure 3 illustrates the effect of using different *α* gains and how *σ* evolves during a second-order system response to a unitary step reference signal. Based on the force error between the force reference and system response and substituting it in expression (4), we can see how the adaptation law modifies *σ* during the transitory and steady state for different values of *α.* Using a very low gain *α* can make almost no difference with a fixed-gain *σ* controller while using a high gain *α* can saturate *σ*. A value of 0.005 is assigned in this illustrative example to $\sigma_{max}$, and it is the value that *σ* approximately takes in the steady state, when the force error is zero.

As stated in (Cao et al., 2020), decreasing the *α* and *β* gains decrease the sensitivity to the force error and the DAAC shows a similar behavior to the AC controller. Care should be taken when tuning the *β* parameter as the noise coming from the force sensor and disturbances can make the derivative term of the force error jeopardize the sigma evolution unless assigning low *β* values or by applying a low-pass filter on the derivative of the force error signal that the *β* multiplies.

#### 3.1.3 Switching Controller

**FIGURE 4:**
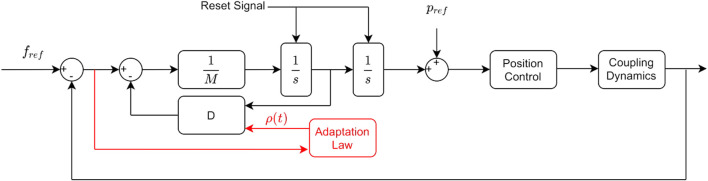
AC diagram (black) and DAAC diagram which shares the same control structure, but with an additional adaptation law (red).

Even with the fast reaction that these controllers have to disturbances, contact losses eventually occur as we have observed in our experiments. Switching to a free motion controller can then be followed until contact is regained. The switching control for the admittance and adaptive admittance is integrated as shown in the control diagram in Figure 4.

A contact or contact loss is determined through force measurement. The control structure remains the same in all the phases of the interaction, the switching between a position and force control is done through the position and force reference signals and a digital reset signal for the two integrators. The settings to change in the controller are different in the contact to noncontact and the noncontact to contact events. The steps that govern the switching between both controllers are given in the following.

Noncontact to contact switching steps:

• The position reference is set constant to its last value before getting into contact.
• The force reference is set to the desired force to apply.

Contact to noncontact switching steps:

• The force reference is set to zero.
• The position reference is initialized using the last reading before the contact was lost.
• The two integrators (Figure 4) are reset to zero.
• For the dynamic adaptive controller, *ρ* is reset to zero.

**FIGURE 5:**
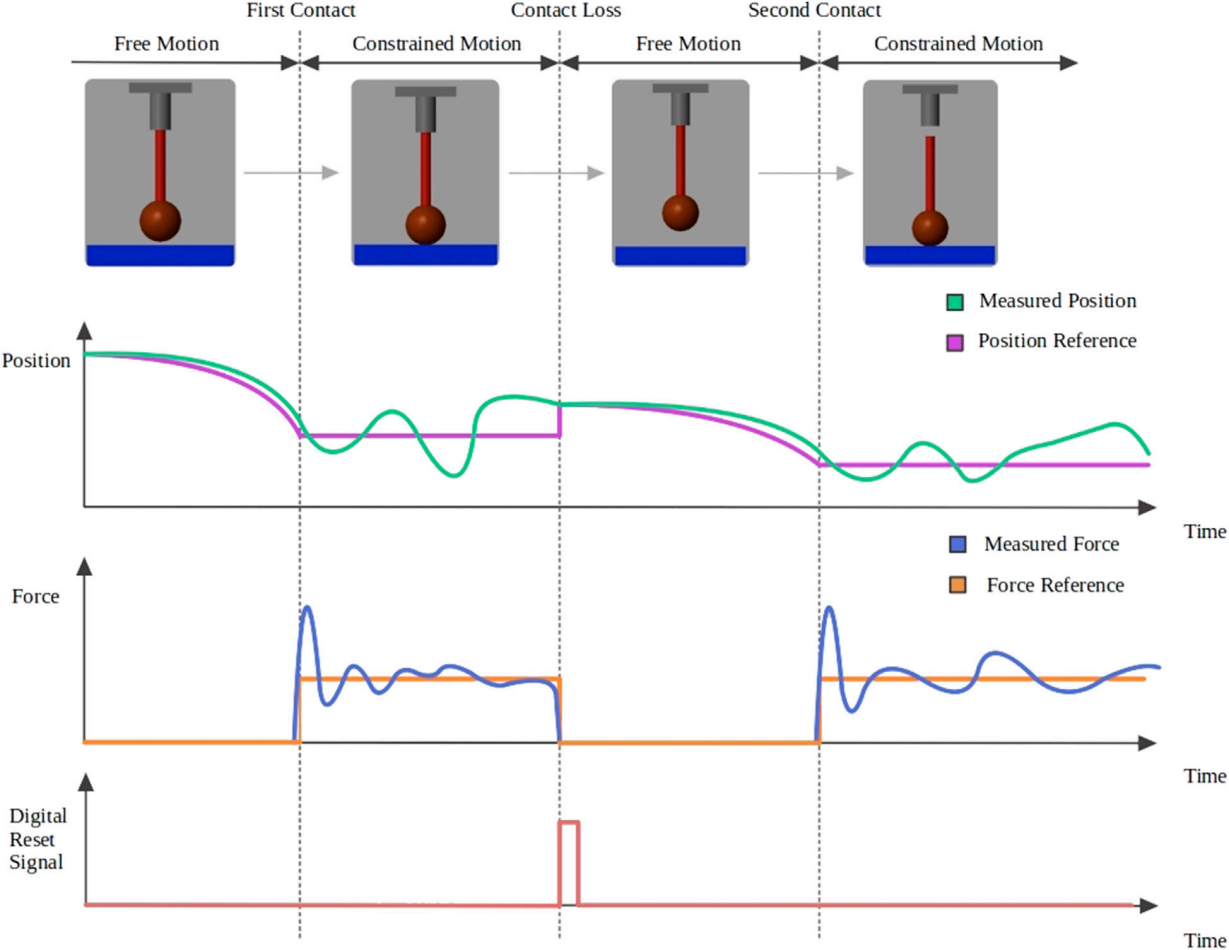
Interaction with a sudden contact loss. Signals plotted are the commanded (purple) and measured (green) position, the commanded (orange) and measured (blue) force, and the reset signal (red) for the two integrators.

**FIGURE 6:**
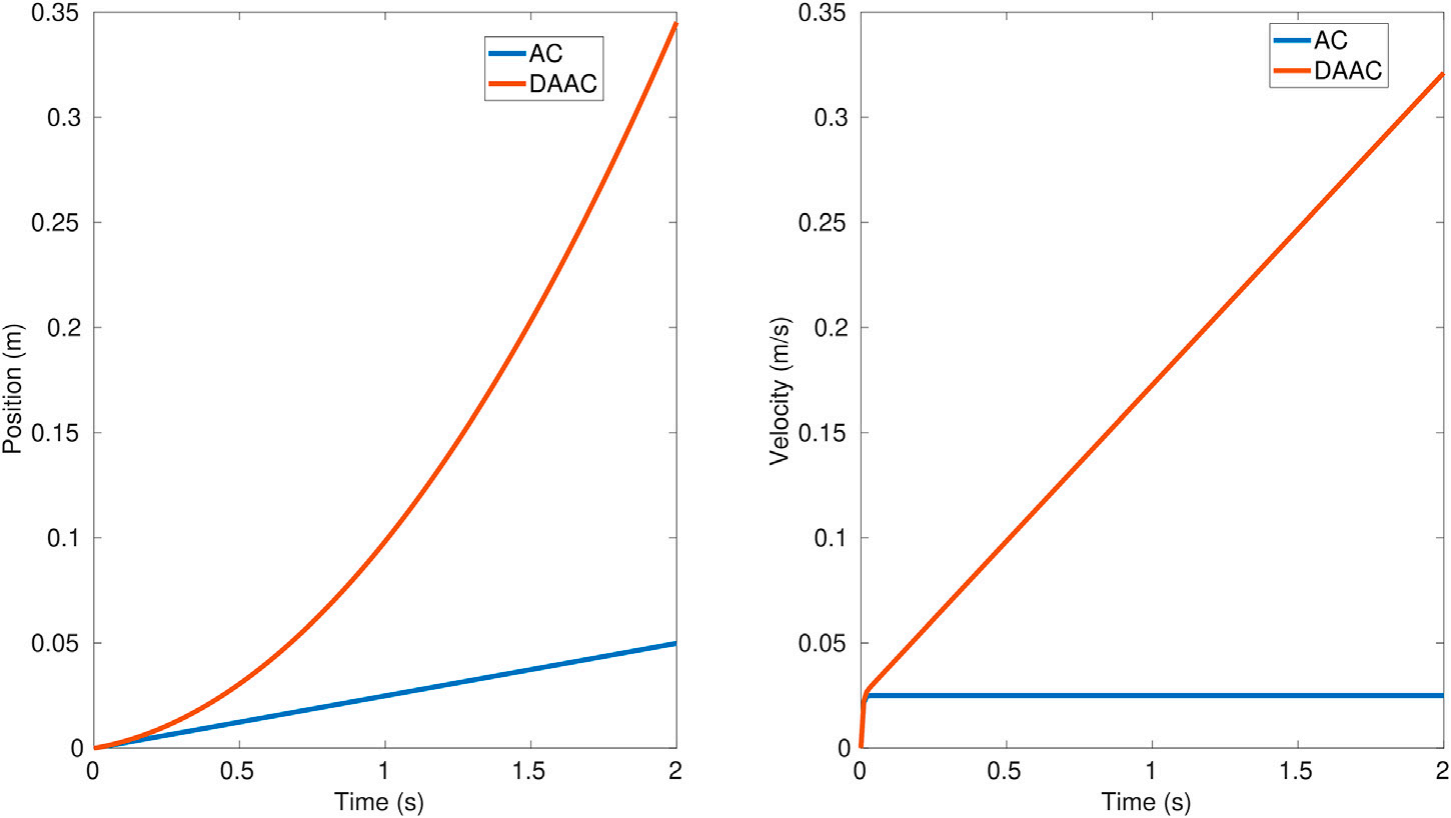
Position and velocity evolution with the AC (blue line) and DAAC (red line), once there is a contact loss and no switching to free motion control.

To better illustrate these switching procedures, a contact loss sample case is shown in Figure 5. The position and force reference (purple and orange signals) in addition to the reset for the integrators (red signal) are the commanded output signals from the switching supervisor, whereas the position and force measurements (green and blue signals) are the inputs. The illustration also shows how the commanded force, position, and reset signals change during the contact and contact loss events. A question that arises is if for brief contact losses it is better to maintain the force control or to make the switching. The answer to that question is clarified in the results section based on the data from the experiments performed. As it has been shown, the switching is not done with a change in the control structure, but by input reference signals from the switching supervisor. If we do not want to switch to a position control when there is a loss of contact, the switching supervisor only has to maintain the commanded references as if nothing had occurred. By solving the differential equations that govern the controllers, we can see the evolution of the end-effector motion dynamics in the force-controlled dimension when the contact is lost and no switching is made. Figure 6 shows the evolution for the AC (blue line) and DAAC (red line) taking the position and velocity set to zero as the initial state. This demonstrates that the AC will move at constant speed in free space, whereas the DAAC will instead accelerate at a constant rate with the consequent accumulation of kinetic energy until contact is made.

Using the switching between controllers allows having a control on the speed of the robot so it does not go beyond a prespecified limit value that prevents dangerous impacts and their side effects: bouncing off, induced oscillations, or damaging the equipment.

In this study, for the free motion control, when the contact is lost, a position-based feed-forwarded velocity control is applied in the local frame of the end effector as expressed in Eq. 6

$${\dot{p}}_{in}\left(t\right)={\dot{p}}_{ref}\left(t\right)+K_{p}\left(p_{ref}\left(t\right)-p\left(t\right)\right)$$ (6)

where $K_{p}$ is the proportional gain matrix, ${\dot{p}}_{ref}\left(t\right)$ is the known sinusoidal reference velocity as a feed-forwarded term, and $p_{ref}\left(t\right)$ is the reference position that is defined as a sinusoidal term. When the contact is lost, the last position of the end effector is set as a constant for the initial position of the reference position $p_{ref}\left(0\right)$.

### 3.2 Control Design for Surface Tracking

It is assumed that the surface of the object that is interacted by UVMS has a 3D shape. As stated before in this study, we have directly adapted our previously published position control method (Moura et al., 2018) for surface tracking. In this method, first, a 2D planar movement is defined with a parametric function of p(q) and a projection of a planar trajectory onto the surface is transformed to the local frame to command the velocity input as

$${\dot{p}}_{in}\left(t\right)=\left[\begin{array}{cc} R & 0\\ 0 & R \end{array}\right]\left(K_{D}^{pc}\frac{\partial p_{d}^{P}\left(q\right)}{\partial q}+K_{P}^{pc}\left(p^{P}-{\hat{p}}_{d}^{P}\left(\hat{q}\right)\right)\right)$$ (7)

where *R* is the rotation matrix of the end-effector frame with respect to the inertial frame of the robot manipulator, $K_{P}^{pc}$ and $K_{D}^{pc}$ are P and D position control gains, respectively, and the parametric functions $p_{d}^{P}\left(q\right)$ and $\frac{\partial p_{d}^{P}\left(q\right)}{\partial q}$ are the desired trajectory tracking inputs. The desired trajectory ${\hat{p}}_{d}^{P}\left(\hat{q}\right)$ is estimated with a parameter $\hat{q}$ in the tangential planar surface frame as $\hat{q}=\text{arg}\underset{q}{\text{ min}}\left\|p^{P}-p_{d}^{P}\left(q\right)\right\|$ to minimize the trajectory error. Please refer to (Moura et al., 2018) for further information.

## 4 Setups

### 4.1 Simulation Setup

The simulations were performed in Matlab Simulink with a simplified 1 DOF linear actuator interacting perpendicularly with a flat environment, as shown in the first row of Figure 5. The reason for using such a simple scenario was to isolate the surface tracking and perpendicular alignment control features of the actual application and to focus only on the switching controller structure when contact takes place and the adaptability required for force tracking on a surface with unknown properties and location. The interaction is modeled as a linear spring-damping system (Kevin-Voigt model) which can represent the coupling dynamics that occur when contact is made with high-stiffness environments where interactions follow a linear behavior as opposed to interactions with nonlinear viscoelastic environments (Flores and Lankarani, 2016). During the simulation, the parameters were set to recreate as much as possible the same conditions of the local perpendicular interaction in the physical setup of interaction experiments.

### 4.2 Physical Experimental Setup

**FIGURE 7:**
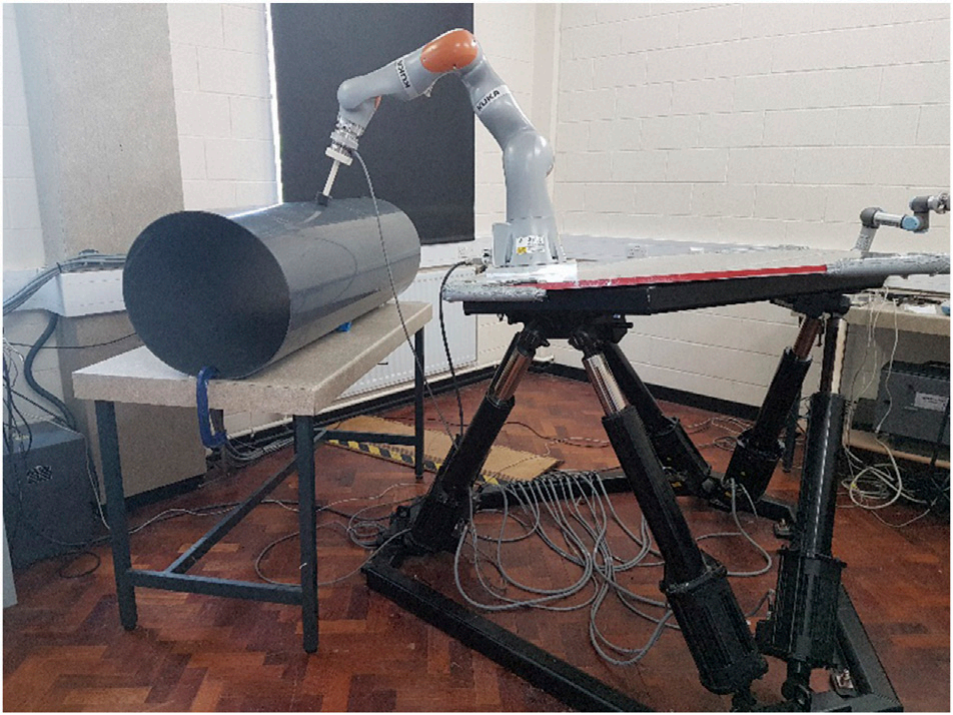
Emulating UVMS *via* using a KUKA IIWA manipulator mounted on a Stewart Platform and interacting with a pipe.

The experimental setup shown in Figure 7 consists of a KUKA IIWA 7 DOF robot manipulator to emulate an underwater manipulator, a 6 DOF Stewart Platform to emulate an underwater vehicle on which the manipulator is mounted, and an ATI Gamma NET FT force sensor attached to the end effector of the manipulator for the force control. A PVC vent pipe with a diameter of 500 mm and a thickness of 4 mm was used to emulate the underwater object that the end effector of the manipulator contacts and interacts with.

## 5 Results

In this section, we present the experimental results with four different controllers: AC, DAAC, and their switching counterparts, switching admittance control (SAC), and switching dynamic adaptive admittance control (SDAAC). The experiments are performed both as simulation in Matlab Simulink and with the physical experimental setup using identical disturbance signals in both cases. The reason to test the controllers in both simulation and physical setup is to verify that the results obtained are consistent and not highly dependent on unknown factors specific to a scenario. Finally, we demonstrate with physical output of the experiments that the DAAC as the ultimate outcome of this study performs better than the AC which was the starting point we had before this study.

In the simulation and experimental studies, three proposed controller methods (DAAC, SAC, and SDAAC) are compared with a standard AC with two different base disturbance effects applied to the base. In the first scenario, a sinusoidal movement is commanded with 0.05sin(2πT/20) on *X*, *Y*, and *Z* directions of the Stewart Platform in the physical setup, whereas only the *Z* direction sinusoidal is applied in the single direction movement in simulation. In the second one, real position data of the Falcon ROV under position disturbances in a water tank is applied on *X*, *Y*, and *Z* directions of the Stewart Platform, and again only the *Z* component of this disturbance is applied in the 1 DOF simulation. While the disturbance in the first scenario provided us with a controlled environment to easily monitor and assess the vehicle motion under each controller, the disturbance in the second scenario provided us with a more realistic evaluation where water dynamics and physical structure of an actual vehicle were reflected in the resulting body motion.

With the physical setup, the end effector of the KUKA manipulator begins to move in *Z* direction in the operational space until it achieved contact with the pipe surface at the desired force level $f_{d}\left(z\right)=-2\text{N}$, and then the end effector performs a raster trajectory on the pipe surface in *X* and *Y* directions of the operational space at the local frame. For all controllers, the generalized inertia and damping coefficients are chosen as M=0.4 and D=80. For the SDAAC, the limit of adaptive gain is chosen as $\sigma_{limit}=0.01$ for the adaptation rule of the dynamic damping parameter *ρ*. For the switching controllers (SAC and SDAAC), in the case of noncontact, a velocity feed-forward augmented PID position controller is applied to the end effector of the manipulator. The feed-forward velocity term is chosen as 0.16πsin(2πT/10) and control gains for PID are $K_{P}=0.3$, $K_{I}=0.0$, and $K_{D}=0.0$.

We used three performance measures to compare the four controllers: the total duration of loss of contact in each experiment, the mean square error of force tracking, and the standard deviation of the force error in Newtons. All the measures across the four controllers with the two forms of disturbances are tabulated in Table 1.

**TABLE 1:** Mean and standard deviations of the squared force errors on Z direction and the total duration of contact loss in between the first contact and the end of the trajectory for four controllers in two application scenarios. Four controllers are compared both in simulation and in the physical experimental set, in each case with an artificial sinusoidal disturbance and realistic underwater vehicle motion disturbance: 1) AC: admittance controller; 2) DAAC: dynamic adaptive admittance controller; 3) SAC: switching admittance controller; 4) SDAAC: switching dynamic adaptive admittance controller.

	Simulation				Physical experiment		
Application scenarios	Control methods	Noncon. dur. (sec)	Mean of Sq.F.Er. (N)	Std. dev. of Sq.F.Er. (N)	Noncon. dur. (sec)	Mean of Sq.F.Er. (N)	Std. dev. of Sq.F.Er. (N)
Sinusoidal base disturbance	AC	6.493	1.930	5.579	3.114	0.913	1.154
	DAAC	5.969	1.944	5.728	0.0	0.399	0.92
	SAC	**1.006**	**1.787**	**5.606**	2.467	0.726	0.918
	SDAAC	1.015	1.836	5.764	**0.0**	**0.211**	**0.311**
Real ROV base disturbance	AC	5.379	1.645	8.037	6.072	4.281	42.17
	DAAC	4.781	1.629	8.043	4.418	0.749	**1.149**
	SAC	**0.767**	1.497	8.023	3.43	1.565	9.271
	SDAAC	0.787	**1.483**	**7.928**	**2.71**	**0.616**	1.181

The bold values indicate the best performance achieved for each evaluation criterion in each of the two scenarios by applying the four different controllers.

Table 1 shows that the last two controllers, SAC and SDAAC, perform superior to the others considering the loss of contact, with both disturbances in simulation and the realistic disturbance with the physical setup. In case of sinusoidal disturbance with the physical setup, SDAAC and DAAC are superior as both eliminate the losses totally; however, SDAAC demonstrates superior performance when also force tracking is taken into account. This shows that switching to a position controller when contact is lost results in a considerable improvement. And although the performance of SAC and SDAAC is similar in the simulations, we observe a significant difference indicating superior performance of the SDAAC with the physical experimental setup. This means that the adaptive feature in this controller is successful to eliminate the unmodeled disturbing effects in the real-physical setup. Overall, the SDAAC controller, which incorporates both switching and adaptivity features, is superior over all other controllers both in simulation and with the physical setup when both loss of contact and force tracking are considered. Furthermore, the standard deviation of the force tracking error indicates that there are no significant spikes in level of force interaction with SDAAC as the values for SDAAC are the minimum in three cases (simulation with both disturbances and physical setup with sinusoidal disturbance) and very close to the minimum in the fourth case (realistic disturbance with physical setup).

**FIGURE 8:**
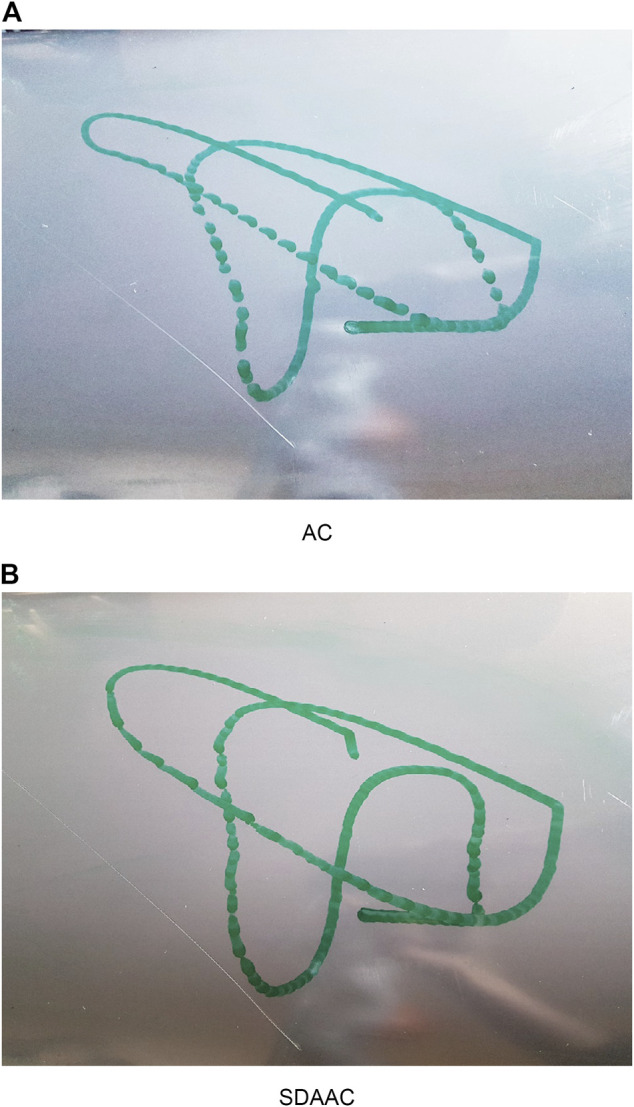
Experimental results (drawings on the pipe) for sinusoidal base disturbance.

**FIGURE 9:**
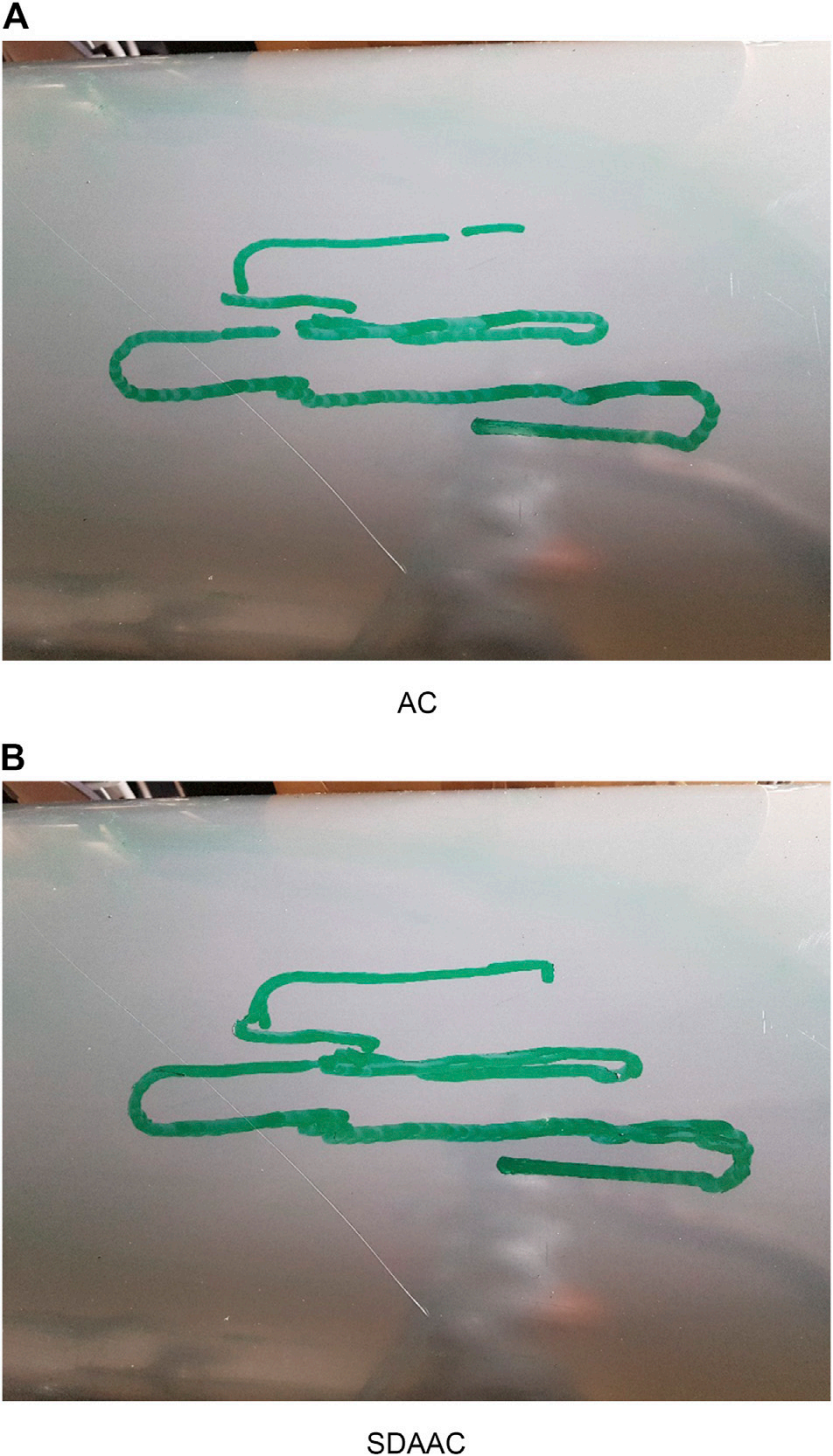
Experimental results (drawings on the pipe) for real ROV base disturbance.

**FIGURE 10:**
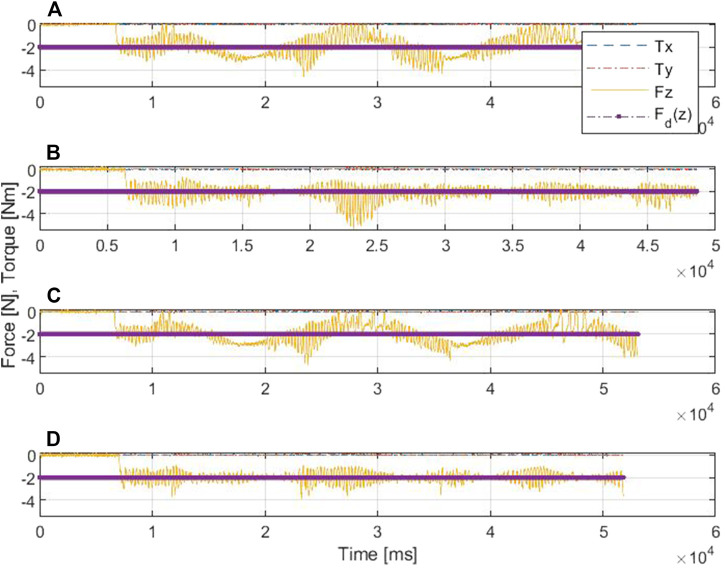
Comparative results of the force/torque measurements in scenario I: **(A)** is for AC, **(B)** is for DAC, **(C)** is for SAC, and **(D)** is for SDAAC.

**FIGURE 11:**
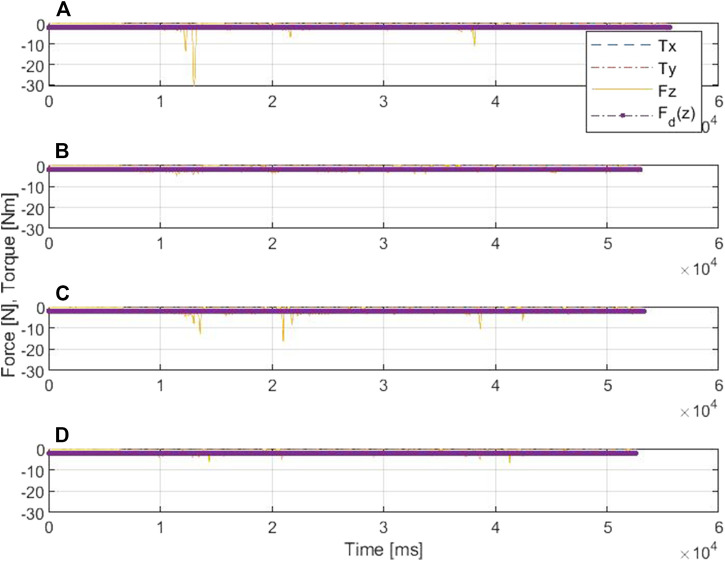
Comparative results of the force/torque measurements in scenario II: **(A)** is for AC, **(B)** is for DAC, **(C)** is for SAC, and **(D)** is for SDAAC.

Figures 8, 9 show the drawing on the pipe made with the marker attached to the end effector of the KUKA manipulator when the base was under sinusoidal and realistic ROV position disturbances, respectively; each figure compares the performance of AC and SDAAC controllers. From the figures, it is clearly observed that the SDAAC controller outperforms the AC controller to obtain a continuous drawing on the pipe, in each case. Figures 10, 11 present a comparison of all four controllers demonstrating the force measured in *Z* direction in the local reference frame of the end effector (perpendicular to the pipe surface) during the physical experiments under the sinusoidal and realistic disturbances, respectively. In the sinusoidal experiment, where there is no or small amount of contact loss, the AC and SAC couple and the DAAC and SDAAC couple demonstrate similar performances as the switching is not much activated. However, we can see that the DAAC and SDAAC have a better disturbance rejection than the AC and SAC, demonstrating the positive impact of the adaptivity. For the ROV disturbance experiment, by comparing the DAAC and SDAAC data, we observe that when there is a contact loss, the impact is handled much better with SDAAC and that prevents high overshoots and oscillations that could create further contact losses.

## 6 Conclusion

In this paper, we have focused on searching for a model-free adaptive controller for maintaining contact with an asset surface with a mobile manipulator system when the mobile base vehicle position is under unknown disturbances. An advantage of having a model-free controller is that it is easily applicable to other platforms than UVMS targeted in this study, such as manipulators attached to aerial, terrestrial, or compliant bases. The controller is required not only to maintain contact with an unknown surface and a desired level of force but also to prevent dangerous impacts and reduce and handle contact losses.

We started with a fixed AC which is passive and has a good behavior at the impact phase for a wide set of conditions. The downside with AC is its slow reaction to overcome disturbances, which makes it more prone to contact losses. In order to make the system faster, we incorporated adaptivity and developed the DAAC which combines the good behavior in the impact phase as the AC and has a better reference following and disturbance rejection. From the beginning, it was clear that, for long contact losses, it is better to switch to free motion control rather than letting AC and DAAC evolve in free space until regaining contact. But in fact, most of the contact losses are very short in duration (many imperceptible), so a question arises if it is better to define a new parameter that will decide the switching when the contact loss is longer than a predefined duration. To answer this question, we developed the switching counterparts of the two controllers as SAC and SDAAC and compared all four controllers in the Matlab simulation and the physical experimental setup to make sure that the results were consistent and not linked specifically to the testing platform.

The experiments have shown that even when there are very brief contact losses, switching to a position controller, rather than maintaining the force control, results in an improvement in terms of contact duration and force tracking accuracy. Among all four controllers, SDAAC, which incorporates both adaptivity and switching features, performed superiorly both in simulation and with the physical setup, considering both measures of loss of contact and force tracking. This result verifies the advantage of adaptivity and switching features in maintaining contact with a structure with a mobile-based manipulator system when the mobile base is under disturbances.

In future work, in order to improve the force tracking for our specific underwater application, we plan to integrate more knowledge in the controller from the vehicle-manipulator motion and coupling dynamics as well as data from additional sensors such as computer vision. This will allow transitioning from a model-free controller with a fast reaction to disturbances strategy to a model-based controller where we can also identify the properties of the environment and apply some predictions.

## Data Availability

The original contributions presented in the study are included in the article/Supplementary Material; further inquiries can be directed to the corresponding author.
